# Digital Phenotyping Methods to Measure or Detect Social Behaviour in Patients With Serious Mental Illness (SMI): A Systematic Review. a Closer Look at Bipolar Disorder

**DOI:** 10.1192/bjo.2022.227

**Published:** 2022-06-20

**Authors:** Samyak Pandey, Aryn Azlan, George Gillett

**Affiliations:** 1 King's College London, London, United Kingdom; 2 Institute of Psychiatry, Psychology & Neuroscience, King's College London., London, United Kingdom

## Abstract

**Aims:**

To provide a fresh insight into the extent digital phenotyping methods have been employed to measure or detect social behaviour in patients with SMIs; with a closer look at those used in Bipolar Disorder (BD); to give findings on the validity, reliability, acceptability and tolerability of these digital phenotyping methods.

**Methods:**

Using specified search terms relating to digital phenotyping metrics and terms related to SMIs, a thorough literature search strategy for studies was employed across the following electronic databases: PubMed, Embase, and PsychINFO - from inception to July 2021.

Included studies employed digital phenotyping methods, collecting either passive, active or mixed-modal data, which in principle reported metrics representing social behaviour on patients with an SMI. Here we present a preliminary analysis of studies reporting results for patients with BD, with a particular focus on tolerability and acceptability.

**Results:**

Of 4,646 records initially screened, a subgroup of 9 studies (n = 474) directly focusing on patients with BD are reported here. Across the studies, we find a modest adherence rate towards these applications by patients, ranging from 72.6% to 89.2%. Methods used by the studies include the frequency of phone calls and text messages, and self-reported and observer ratings of social and interpersonal functioning. The collection of such digital phenotyping data appears tolerable and acceptable to participants with BD, with patients reporting them to be supportive and only mildly intrusive.

**Conclusion:**

Our preliminary analysis suggests that digital phenotyping of social behaviour may be acceptable and tolerable to participants with Bipolar Disorder. In an increasingly digital world, digital phenotyping methods of social behaviour may assist physicians with clinical assessment and prediction of clinical outcomes including relapse. Future analyses will assess the reliability and validity of the data that such methods yield, and their potential therapeutic value.

